# An Eastern County from an European Eastern Country—The Characteristics of Cutaneous Microbiome in Psoriasis Patients—Preliminary Results

**DOI:** 10.3390/life14060678

**Published:** 2024-05-24

**Authors:** Diana Sabina Radaschin, Alina Viorica Iancu, Alexandra Mariana Ionescu, Gabriela Gurau, Elena Niculet, Florin Ciprian Bujoreanu, Florentina Nastase, Teodora Radaschin, Liliana Gabriela Popa, Roxana Elena Axente, Alin Laurentiu Tatu

**Affiliations:** 1 Department of Dermatology, “Saint Parascheva” Infectious Disease Clinical Hospital, 800179 Galati, Romania; diana.radaschin@ugal.ro (D.S.R.); alin.tatu@ugal.ro (A.L.T.); 2 Department of Clinical Medical, Faculty of Medicine and Pharmacy, “Dunarea de Jos” University of Galati, 800385 Galati, Romania; 3 Multidisciplinary Integrated Centre of Dermatological Interface Research (MICDIR), “Dunarea de Jos” University of Galati, 800385 Galati, Romania; 4 Department of Morphological and Functional Sciences, Faculty of Medicine and Pharmacy, “Dunarea de Jos” University, 800385 Galati, Romania; alina.iancu@ugal.ro (A.V.I.);; 5 Faculty of Sciences and Environment, “Dunarea de Jos” University, 800385 Galati, Romania; 6 Department of Neuropsychomotor Rehabilitation, “Sf. Ioan” Clinical Hospital for Children, 800487 Galati, Romania; 7 Radiology Department, Fundeni Clinical Institute, 022328 Bucharest, Romania; 8 Dermatology Department, Carol Davila University of Medicine and Pharmacy, 050474 Bucharest, Romania; 9 Faculty of Medicine and Pharmacy, “Dunarea de Jos” University of Galati, 800385 Galati, Romania; elena.axente@ugal.ro

**Keywords:** psoriasis, skin microbiome, cutaneous microbiome, inflammatory skin disease

## Abstract

The cutaneous microbiome represents a topic of high interest nowadays. Multiple studies have suggested the importance of the skin microbiome in different dermatological pathologies, highlighting the possible implications of cutaneous microorganisms in either the pathogenesis or prognosis of skin maladies. Psoriasis represents a common inflammatory skin disease, with a high prevalence in the worldwide population. The role of the cutaneous microbiome in psoriasis could explain a number of pathogenic theories and treatment objectives of this incurable skin disease. Our interest in the characteristics of the cutaneous microbiome, especially in psoriatic patients who attended a tertiary dermatological centre in Galati, Romania, is reflected in our current study, of which the preliminary results are discussed in this article. Using three types of skin sampling techniques (swabs, adhesive tape, and punch biopsies), we tried to characterise the microorganisms harboured in the skin of psoriatic patients and healthy individuals. This study was performed using culture-based probes, which were analysed using MALDI-TOF mass spectrometer equipment. Our preliminary results suggested that the greatest diversity was observed in the perilesional areas of psoriatic patients. The lowest cutaneous diversity was obtained from sampling psoriatic plaques. These results are similar to other studies of the cutaneous microbiome in psoriasis. The most frequent microorganisms found in all groups studied were of the *Staphylococcus* species: *Staphylococcus epidermidis*, *Staphylococcus hominis*, and *Staphylococcus aureus*. Analysing the living environment of each individual from this study, our preliminary results suggested different results from other studies, as higher diversity and heterogenicity was observed in urban environments than in rural living areas. Regarding the differences between sexes, our preliminary results showed higher quantitative and qualitative changes in the skin microbiome of male participants than female participants, opposite to the results found in other studies of the cutaneous microbiome in psoriasis. Given these preliminary results, we can conclude that we have found important differences by studying the cutaneous microbiome of psoriatic patients and healthy control individuals from a population that, to our knowledge, has not been yet studied from this point of view. Our results showed important characteristics of the skin microbiome in an Eastern European population, where cultural and environmental living habits could influence the cutaneous microbiome.

## 1. Introduction

Psoriasis is a chronic, multifactorial, inflammatory disease which is characterized by genetic predisposition and unique autoimmune traits. It affects approximately 2% of the worldwide population and is a common cause of low quality of life index in patients suffering from this disease [1,2]. The chronic inflammatory cascade involved is triggered by exogenous or endogenous factors, resulting in the hyperproliferation of keratinocytes and thickening of the epidermis and dermis, as well as the abnormal infiltration of immune cells with proinflammatory effects. Psoriasis is a multisystem disorder affecting not only the skin, but also the joints, the cardiovascular system, and the central nervous system. The most common clinical form of psoriasis, psoriasis vulgaris, is characterized by well-circumscribed, indurated, erythematous papules or plaques with a typical scaly surface, affecting predominantly and symmetrically the elbows, knees, and scalp [1,2].

As a worldwide-prevalent skin disease, psoriasis can affect people of various ages, ranging from 16 to 60 years old. It depends on multiple external factors, with a primary chronic involvement of the skin and joints, and secondary involvement of the mucosal membranes and nails. The course of this multisystem disorder is usually unpredictable, with periods of acute flare-ups followed by periods of remission of an unknown length [1,2].

Historically, psoriasis has been described from the early ages. The first clinical description of psoriasis has been attributed to Aurelius Cornelius Celsus (25–45 AD), who designated erythematosquamous lesions of the hands as “*impetigo*”. Later, Galen (131–201 AD) used the term psoriasis to describe a pruritic and squamous eruption of the eyes and genitals. In the Middle Ages, a mistranslation of Arabic texts lead to the confusion that all squamous disorders were linked to *leprae*; therefore, psoriasis suffering patients were considered for a very long time to be contagious and were excluded from society. They had to announce their presence by wearing bells attached to recognizable clothes. They were not allowed to talk to or touch healthy individuals. It was not until 1808 that Robert Willian, the father of modern dermatology, described the disease “*psoriasis difussa*” in relation to occupational disease. He presented a distinctly defined description of psoriasis and its clinical forms [3,4].

Nowadays, psoriasis is considered to be a rather common inflammatory disease. Its pathogenicity and therapeutical approaches represent subjects of high interest in the medical field, but even though many modern advances have been made in psoriasis research, the disease continues to negatively impact the quality of life of patients.

The pathogenesis of psoriasis implies an inflammatory cascade triggered by external or internal factors leading to hyperproliferation of keratinocytes, accelerated cellular turnover, and complex interactions between innate and adaptive immune cells. The cause of the disease remains unclear, but studies have shown that an exacerbation of psoriasis may be triggered by external agents. Stress, trauma, drugs, or pathogens can induce a flare-up in the course of the disease. In children, acute guttate psoriasis can develop following an upper respiratory tract infection. Subsequent to streptococcal pharyngitis, given the similitude between streptococcal M protein and keratin 17, T cells proliferate and initiate proinflammatory responses, leading to clinical lesions of guttate psoriasis [5,6]. Given this well-known association between external factors and the exacerbation of psoriasis, in genetically predisposed individuals, the role of the cutaneous microbiome in pathogenesis and its implications in psoriasis are becoming a subject of great interest.

The cutaneous microbiota is defined as a community of commensal, symbiotic, and pathogenic microorganisms that are harboured by the human skin. The cutaneous microbiome refers to the genomic analysis of cutaneous microorganisms [7,8]. It is a complex ecosystem in which bacteria, archaea, fungi, and viruses become essential to skin physiology and immunity. Commensal microorganisms are usually found on the surface of the skin and are considered to be harmless. Transient microorganisms arise due to environmental instabilities and persist for a limited amount of time [6,9,10]. Currently, there are about 200 microorganisms known as pathogenic [7].

In the case of psoriasis, prior research has thoroughly investigated the dysbiosis of the cutaneous microbiome and the role of microorganisms in the pathogenesis of this chronic inflammatory disease. Research on the human microbiome has recently been improved by the use of genomic testing, which is based on the analyses of 16 s ribosomal ARN [11]. The diversity of the skin microbiome consists of bacteria from the *Firmicutes*, *Actinobacteria, Proteobacteria,* and *Bacteroidetes* families [8]. The genera usually found on human skin are Propionibacterium, *Streptococcus*, *Staphylococcus*, *Corynebacterium*, and Lactobacillus [11].

## 2. Materials and Methods

Our study was conducted between May and July 2023 and included a total of 33 subjects aged from 21 to 73 years old. Written consent from the patients and control group was provided. The approval of the Ethical Committee of “Saint Parascheva” Clinical Hospital of Infectious Diseases, Galati, Romania and the Ethical Committee of the Medical College of Galati was also obtained.

This study was performed on a total of 33 participants, comprising 20 individuals diagnosed with psoriasis and 13 healthy controls. The demographic characterisation of the studied groups is presented in Table 1.

Among the control group, patients with inflammatory skin diseases or neoplasms, and individuals with first-degree relatives diagnosed with psoriasis or psoriatic arthritis, were excluded. Patients undergoing treatment with topical or systemic antibiotics, immunomodulators, or probiotics were also excluded from this study. The participants were permitted to use only mild soaps and emollients 2 weeks prior to the start of sampling, and were advised not to take a shower in the 24 h immediately before sampling. The patients diagnosed with psoriasis (clinical and histopathological) were assessed using the Psoriasis Area and Severity Index score (PASI) and the Dermatology Life Quality Index (DLQI).

The cutaneous microbiota was analysed using biological samples from the skin of the participants. The samples collected from the psoriatic group were divided into three categories: swabs, tape stripping, and skin biopsies. Regarding the healthy control group, samples included swabs and tape stripping only. Tape stripping samples were obtained using sterilised adhesive stripes 2 × 4 cm in size, applied directly onto the skin. The cutaneous biopsies were performed after obtaining temporary analgesia using intradermal 1% lidocaine, using a 3 mm biopsy punch. No antiseptic solution was used.

Furthermore, all samples were inoculated on both aerobic and anaerobic mediums. A total of 12 samples per patient were collected, comprising four tape stripping samples (two from lesions and two from non-lesion sites), four swab samples (two from lesions and two from non-lesion sites), and four punch biopsies (two from lesions and two from non-lesion sites).

The sampling sites were chosen from psoriatic plaque lesions and non-lesional skin. The different characteristics of the skin microbiome were regarded, and we obtained samples from both moist and dry body areas, symmetrically when possible. The healthy control group sampling included only tape stripping and swabs from body areas similar to those taken from the psoriatic group.

### 2.1. Isolation and Identification of Microorganisms

After performing the skin sampling, the anaerobe probes were inoculated on fluid thioglycollate medium (Thermo Fischer Scientific, Basingstoke, UK, Oxoid Ltd.), and the aerobe samples were inoculated on glucose nutrient broth (Thermo Fischer Scientific, Basingstoke, UK, Oxoid Ltd.), necessary for the growth of microorganisms. The bacterial colonies were sub-cultured onto agar with 5% sheep blood (Thermo Fischer Scientific, Basingstoke, UK, Oxoid Ltd., Basingstoke, UK) and incubated for 24 h at 37 °C in aerobic and anaerobic conditions in the Laboratory Medicine Department of “Saint Parascheva” Clinical Hospital of Infectious Diseases, Galati, Romania. Identification of bacterial isolates was carried out in the Clinical Laboratory of the Paediatric Hospital of Galati using MALDI-TOF with an Auto MS 100-Mass Spectrometer (Autobio Diagnostics Co., Ltd., Zhengzhou, China).

### 2.2. Quality Control

Reference strains for quality control were used in the isolation and identification process, such as: ATCC 19615—*Streptococcus pyogenes*; ATCC 25923—*Staphylococcus aureus*; ATCC 25922—*Escherichia coli*; ATCC 27853—*Pseudomonas aeruginosa;* and ATCC 10231—*Candida albicans* (Thermo Fisher Scientific, Lenexa, KS, USA)

### 2.3. Statistical Analysis

The statistical analysis was performed in SPSS 29.0. The qualitative data were reported as absolute frequencies and percentages, and the quantitative data were reported as averages and standard deviations. The associations between qualitative data were investigated using the Chi-squared and Fisher tests. Values of *p* < 0.05 were evaluated as statistically significant, and values of *p* < 0.01 were evaluated as statistically highly significant.

## 3. Results

We present the preliminary results of our study regarding the characterization of the cutaneous microbiota in psoriasis.

We performed cutaneous sampling from all participants using three methods: swabs, adhesive taping, and punch biopsies. Each of the samples was then cultured in aerobic and anaerobic mediums. The active group, the psoriatic patients, were analysed using all three methods, but not all patients consented to the biopsy method; thus, a total of thirty-two samples were collected from eight patients diagnosed with psoriasis (four samples from each patient: two from the psoriatic plaques, used for the aerobic and anaerobic mediums, and two peri-lesional samples, one for the aerobic medium, and one for the anaerobic medium). Each individual from the psoriatic group was assessed using psoriatic severity scores and Dermatology Life Quality Index (DLQI). The cutaneous microbiome was analysed in the control group by skin sampling using swabs and adhesive tape.

For each individual in this study, we also established independent variables, such as gender, age, living environment, and diagnosis.

The preliminary results of this study were as follows:

1. The psoriatic group represented 60.6% of the total number of participants, comprising 15 males and 5 females. The control group made up 39.4% of the participants, comprising 10 females and 3 males.
2. The age of the participants was similar across both groups. The mean age in the psoriatic group was slightly higher than that of the control group, with a mean age of 53 in the psoriatic group and 46 in the control group.
3. Regarding the living environment of the participants analysed in our study, participants living in urban environment represented the majority in both groups. A total of 55% of the psoriatic group lived in the city, and 45% in rural areas.
4. PASI scores varied between 2 and 49, with a mean of 18.94. Male participants showed higher average scores than female participants, with a mean of 20.4 versus 14.5, respectively. In addition, the patients living in rural areas had higher PASI scores than those living in urban areas.
5. Regarding the DLQI score, the mean score of the group was 15.5, ranging from 2 to 28. The score was higher for female patients, with a mean of 19.40 versus 14.20 for male individuals. Regarding living environments, those living in rural areas had higher scores than those living in the city.
6. Swab sampling from psoriatic plaques was performed across nine different body zones. The most frequently sampled zones were dry zones, such as the elbows and anterior trunk.

The results showed a higher diversity of microorganisms in the aerobic medium (11) than in anaerobic medium (8). The most frequent species developed on the aerobic medium were *Staphylococcus epidermidis* and *Staphylococcus hominis* (each comprising 30%), as seen in Table 2. In the anaerobic medium, the most frequent species developed were also *Staphylococcus epidermidis* and *Staphylococcus hominis*. *Staphylococcus aureus* was identified in two cases in the aerobic medium, and three cases in the anaerobic medium.

7. Swabs from non-lesional body zones were collected from eight different body areas. The most frequent swabs were taken from moist zones, such as the axillary and laterocervical regions. Seventeen different species were present in the aerobic medium, and nine species were present in the anaerobic medium. The most frequent microorganisms were *Staphylococcus hominis* and *Staphylococcus epidermidis*, as shown in Table 2.
8. In the control group, the majority of swab sampling was also performed in the axillary and laterocervical areas, and we found that there was less diversity regarding bacterial species in this group. Twelve bacterial species developed in the aerobic medium, and eight species on the anaerobic medium. The most frequent species were again *Staphylococcus hominis* and *Staphylococcus epidermidis,* as shown in Table 3.

9. The preliminary analysis regarding the living environments of participants revealed higher diversity and heterogenicity in urban areas than in rural areas. The majority of the species identified in both groups were *Staphylococcus epidermidis*, *Staphylococcus hominis*, and *Staphylococcus aureus*. In participants from an urban environment, our analysis showed the presence of *Acinetobacter* spp., *Bacillus* spp., *Enterococcus* spp., and *Pseudomonas* spp., which were not present in participants from a rural environment, as shown in Table 4.

10. Regarding the gender differences observed in the psoriatic group, as shown in Table 5, swab sampling from psoriatic plaques evidenced a greater number of species and greater diversity in male than in female participants. However, more thorough research on these differences is required, given the small number of participants in this study.

The results from adhesive tape sampling and punch biopsy samples are still under observation. Furthermore, the characterisation of the different species developed on the culture mediums is being studied. The impact of these species on the treatment and development of new plaques is also under observation. The diversity of the cutaneous microbiome in our study is the object of further study.

## 4. Discussion

The pathogenic mechanisms related to psoriasis development have been a subject of great interest over the years, resulting in novel therapeutic approaches for psoriasis. The importance of continuously studying the pathogenic pathways and possible triggers of psoriasis is revealed by the possibility of developing new therapies for the disease, resulting in improvements to the quality of life of patients and achieving control of the disease.

In our study, we aim to characterise the cutaneous microbiota found in patients and control individuals, leading to further development of the pathogenic hypothesis concerning the exogenous triggers of psoriasis, and the implications of pathogenic agents in the course of the disease.

In genetically predisposed individuals, the development of psoriasis is thought to start from exogenous factors such as microbial agents, trauma, or medication, which induce keratinocytic injury [12,13,14]. The aggressed keratinocytes will produce certain molecules, such as antimicrobial peptides (LL-37), which act as antigens and are able to activate antigen presenting cells (APC) like plasmacytoid dendritic cells and natural killer cells. The activation and proliferation of effector T cells is then possible due to migration of activated myeloid dendritic cells into the lymph nodules by tumour necrosis factor (TNF alpha), interferon alpha, and interferon gamma, secreted by activated pDC. Activated naïve T cells migrate via the bloodstream to the skin, and due to proinflammatory cytokines (TNF alpha, IFN gamma, IL-22, and IL-17), they differentiate into LTh1 and LTh17, which are able to secrete proinflammatory cytokines as well. Therefore, a vicious cycle in the pathogenesis of psoriasis is created, and the course of the disease is maintained [14,15,16,17].

The role of the cutaneous microbiome in triggering psoriasis has been studied in recent years [12]. A link between streptococcal infections of the upper respiratory tract and exacerbations of guttate psoriasis has been established [5]. A structural analogy between cytokeratin K14, expressed by the keratinocytes of the stratum basale, and the M protein of streptococcal origin, has been found. T lymphocytes react to both the M protein and K14 cytokeratin, causing an exacerbation in psoriasis. This suggests that these lesions can occur as a result of an exaggerated immune response against pathogens [17]. In the study of Zakostelska et al., performed on mice which received systemic antibiotic therapy, a deficient microbiome led to lower frequencies of proinflammatory cells such as LTh17, which are known to have major roles in the inflammatory cascade of psoriatic disease [18]. Therefore, further studies are needed to determine the exact impact of topical or systemic antibiotic therapy in the inflammation process and course of psoriasis. In another study, new psoriatic plaque formation was observed when applying suspensions of *Malassezia ovalis* to the unaffected skin of psoriasis patients. Such reactions to *Malassezia* were observed after the indirect submission of fungi from the scalp onto the skin of patients with psoriasis [19]. In all of these cases, improvement in psoriatic lesions was observed after systemic antifungal treatment [19].

The skin and its microbiome are important factors in setting an interface between the immune system and the environment [20]. As an example, *Staphylococcus epidermidis* plays a major role in protecting against the agent *Leishmania major*, with the help of a T-cell-mediated response. The inflammation found in psoriasis may be the result of dysregulated immune responses implicating the microbiota; this is supported by the modified microbial profile found in such patients [21,22].

Commensal microorganisms are essential in maintaining skin integrity. Firstly, they block the adherence of pathogens, as shown in the case of *Staphylococcus epidermidis*, which can inhibit the biofilm formation of *Staphylococcus aureus* [22], and by *Corynebacterium acnes*, which restricts the colonization of the skin by methicillin-resistant *Staphylococcus aureus* [23]. Secondly, the immune system can be influenced by the cutaneous microbiome, especially via the modulation of the innate immune response, and particularly through an increase in the secretion of interleukin 2 and interleukin 17, mediated by T-helper lymphocytes [24]. As a consequence, alterations to the skin microbiome may be considered key points in the development and pathogenesis of several chronic inflammatory diseases, such as psoriasis, atopic dermatitis, rosacea, and vitiligo, and may be induced by various treatments [25,26,27].

Regarding the preliminary results of our study, we aimed to characterise the cutaneous microbiome of psoriatic patients and the control group by detecting both qualitative and quantitative differences. The diversity and heterogenicity of the skin microbiome have been evaluated in multiple studies over the years. Prior research has investigated the diversity of bacteria found on psoriatic lesions [27,28,29]. The predominance of *Staphylococcus aureus* was described both in lesional and non-lesional skin. In comparison to healthy individuals, in psoriasis patients, lower levels of *Staphylococcus epidermidis* and *Cutibacterium acnes* were observed [29]. A decrease in *Cutibacterium acnes* levels in psoriatic lesions has also been mentioned in several past studies [30,31].

More recent studies have detected a decrease in the cutaneous microbiome of psoriasis patients compared with healthy individuals [32,33]. Psoriatic lesions have been found to have enhanced levels of *Streptococcus*, *Staphylococcus, Bacterioidetes*, and *Firmicutes* families, while the presence of *Cutibacterium* and actinobacteria was found to be significantly reduced [32,33]. Clinical improvement has been observed after oral probiotic treatment in psoriatic patients [32]. Some studies have shown an increase in *Xanthomandaceae*, a keratolytic bacteria, after clinical improvement in psoriatic lesions post-balneotherapy management [32].

The results we have obtained so far regarding swab sampling outline some important differences between the studied groups in terms of diversity and heterogenicity.

Regarding quantitative modifications in the cutaneous microbiome, a total of 65 different species were identified using swab sampling for all studied groups. Swabs collected from psoriatic plaques revealed the growth of 19 species; peri-lesional sampling revealed 26 species harboured on the skin, and 20 species were found on the cutaneous areas examined in healthy individuals. So far, the greatest diversity has been observed in the peri-lesional areas of psoriatic patients. Sampling from the psoriatic plaques revealed the lowest cutaneous diversity of microbial agents, even lower than that of healthy control group. The control group showed higher diversity than swabs collected from psoriatic plaques and lower diversity than those taken from peri-lesional sampling. These results are similar to other studies, which have suggested lower prevalence of microbial communities in psoriatic plaques [34,35].

Considering the qualitative changes observed in the swab sampling results, in all three groups, the most frequent bacterial species identified were *Staphylococcus epidermidis*, *Staphylococcus hominis*, and *Staphylococcus aureus*. Fungal infections, and the highest number of *Staphylococcus aureus* organisms, were noted in peri-lesional areas. *Cutibacterium acnes* was also observed in peri-lesional skin. The highest number of pathogenic agents, such as *Escherichia coli*, *Enterococcus* spp., and *Klebsiella* spp., were observed in the peri-lesional samples.

The control group showed fewer bacterial species than the psoriatic patients, mostly *Staphylococcus* spp. and *Bacillus* spp. The psoriatic group expressed lower diversity than the other two groups. The main microorganisms developed on psoriatic plaques, in our study, were of the *Staphylococcus* spp. These results were similar to multiple studies that have suggested that colonization with *Staphylococcus aureus* is greater in psoriatic skin than in healthy individuals [36]. This colonization is thought to induce further inflammatory cascade by the ability of T cells to recognize *Staphylococcus aureus* strains through IFN gamma and to maintain an elevated inflammatory response [37].

Analysing the living environment of individuals, the cutaneous microbiome varied depending on rural or urban conditions. The microbiome of individuals living in urban areas did not show high levels of diversity like those living in rural environments. In urban areas, the main source of microbiological agents is represented by human contact, whereas in rural environments, the cutaneous microbiome is influenced by animal contact. The decrease in diversity found in urban areas might be associated with hygienic habits, such as the use of hand sanitizers more frequently. The skin microbiome in rural conditions is characterised by a high level of variability. People working in farms show a higher diversity of cutaneous microorganisms, resembling the environment they work in [38,39,40].

In our study, the preliminary analysis regarding the living environment of each individual revealed higher diversity and heterogenicity in those from urban areas than in those from rural areas, which opposes what most studies have suggested. The majority of species identified in both groups were *Staphylococcus epidermidis*, *Staphylococcus hominis*, and *Staphylococcus aureus*. In participants from urban environments, our analysis showed the presence of *Acinetobacter* spp., *Bacillus* spp., *Enterococcus* spp., and *Pseudomonas* spp., which were not present in those from rural environments, as shown in Table 4.

Therefore, samples collected from psoriatic plaques showed more quantitative changes in those from urban areas than from rural environments. *Staphylococcus aureus* colonisation on psoriatic plaques was more frequent in urban-living patients than rural-living patients. *Acinetobacter* spp. were found on psoriatic plaques in patients living only in urban environments. The psoriatic plaques of patients living in rural areas most frequently revealed colonization with S. *epidermidis* and *S. hominis*.

Regarding the peri-lesional swab sampling, higher numbers of species were observed than in those samples from psoriatic plaques. Individuals living in rural areas most frequently revealed peri-lesional colonization with *S. hominis*, S. *epidermidis*, and *S. aureus*. Other microorganisms found on the peri-lesional sites of patients living in rural areas were *Candida auris*, *Corynebacterium* spp., *E*. *coli*, and *Bacillus* spp. Peri-lesional findings in patients living in urban areas revealed most frequent colonization with *S. epidermidis, S. haemolyticus,* and *S. hominis*. Other microorganisms found were *Cutibacterium acnes*, *Enterococcus* spp., *Klebsiella* spp., and *Streptococcus* spp.

Multiple studies performed in healthy individuals regarding the differences between male and female cutaneous microbiomes have suggested higher numbers of microorganisms in female participants than in male participants [41,42,43]. The preliminary results of our study on psoriatic patients provided opposing evidence. Both samples taken from psoriatic plaques and peri-lesional sites showed a greater abundance of microorganisms in male patients, as shown in Table 5.

Upon analysing the preliminary results from the swab sampling probes, male participants revealed a greater number of species and greater diversity than female participants, both from psoriatic lesions and peri-lesional sampling. Swabs collected from psoriatic plaques revealed less species than those taken peri-lesionally. In swabs from psoriatic plaques, male individuals showed higher diversity and more quantitative changes than female individuals. *Staphylococcus epidermidis* was the most frequent species found on lesional swabs in both male and female participants. *Staphylococcus aureus* was more frequent in psoriatic plaque samples taken from females than males. Peri-lesional swabs also revealed greater diversity in male participants than female participants. *Staphylococcus hominis* and *epidermidis* were the most frequent species found both in males and females. In female participants, *Cutibacterium acnes* and *Streptococcus* spp. were also observed in peri-lesional areas, whereas in male participants, *Pseudomans* spp., *Klebsiella* spp., *Enterococcus* spp., *Corynebacterium* spp., *Bacillus* spp., and *Candida* were observed. These preliminary results might be explained by the hygienic and skin hydrating customs of female patients. The constant use of emollients and exfoliating products by female individuals, especially those diagnosed with psoriasis, could impact the diversity of the cutaneous microbiome. Studies show that the immune system is affected by hormonal changes, and the microbiome is also influenced due to these changes [43].

The limitations of our study include the small number of individuals involved, and the fact that it was limited to a single-centre study. In order to confirm our results further, studies on a larger population should be performed.

## 5. Conclusions

The importance of interactions between the cutaneous microbiome and the immune system stems from the fact that they may generate important data concerning chronic inflammatory diseases. Answers regarding the pathogenesis of psoriasis could be found by studying the cutaneous microbiome and understanding how normal and pathological flora affect inflammatory responses in certain chronic diseases.

This could imply new approaches in the therapeutic management of dermatological diseases such as psoriasis, atopic dermatitis, and acne vulgaris, and possibly increasing the lengths of time spent in remission.

Studying the cutaneous microbiome could reveal more opportunities for research, and perhaps more valuable therapeutic options in the future.

Given these preliminary results, we can conclude that we have found important differences by studying the cutaneous microbiome of psoriatic patients and healthy control individuals from a population that, to our knowledge, has not been yet studied from this point of view. Our results show important characteristics of the skin microbiome in an Eastern European population, where cultural and environmental living habits could influence the cutaneous microbiome.

## Figures and Tables

**Table 1:** Demographic characterisation of the studied groups.

		ACTIVE			CONTROL		
		N	%	N	%	N	%
Gender	Male	15	75.0%	3	23.1%	18	54.5%
	Female	5	25.0%	10	76.9%	15	45.5%
Environment	Urban	11	55.0%	10	76.9%	21	63.6%
	Rural	9	45.0%	3	23.1%	12	36.4%
Total		20	100.0%	13	100.0%	33	100.0%
Age		Mean	Std Error of Mean	Std Deviation	Min	Max	Mean
Active Group		53.70	2786	12,461	21	73	53,50
Control Group		46.92	3661	13,200	27	72	47.00
Total		51.03	2262	12,994	21	73	51.00

**Table 2:** Swab sampling from psoriatic plaques.

Swab Sampling from Psoriatic Plaques	AEROBIC		ANAEROBIC	
	n	%	n	%
Lack of growth	1	5.0	2	10.0
Unidentified	1	5.0	3	15.0
*Acinetobacter* spp.	1	5.0		
*Bacillus subtilis*	2	10.0		
*Enterococcus faecium*	1	5.0		
*Micrococcus luteus*	1	5.0		
*Proteus mirabilis*	1	5.0	1	5.0
*Pseudomonas luteola*			1	5.0
*Staphylococcus aureus*	2	10.0	3	15.0
*Staphylococcus capitis*	2	10.0	1	5.0
*Staphylococcus cohnii*	1	5.0		
*Staphylococcus epidermidis*	6	30.0	7	35,0
*Staphylococcus haemolyticus*			2	10.0
*Staphylococcus hominis*	6	30.0	4	20.0
*Staphylococcus lugdunesis*			1	5.0
*Staphylococcus warneri*	1	5.0		
Total	11		8	

**Table 3:** Swab sampling from the peri-lesional (active) and control group: bacterial species identified on aerobic and anaerobic medium.

Swab Sampling		Aerobic		Anaerobic	
		n	%	n	%
Active Group					
	Lack of growth	-		1	5.0
	Unidentified	-		2	10.0
	*Bacillus anthraci*	1	5.0		
	*Bacillus methylotrophicus*	1	5.0		
	*Bacillus siamensis*	1	5.0		
	*Candida auris*	1	5.0		
	*Corynebacterium aurimucosum*	1	5.0		
	*Cutibacterium acnes*			1	5.0
	*Enterococcus avium*	1	5.0		
	*Escherichia coli*	1	5.0		
	*Exiguobacterium aurantiacum*	1	5.0		
	*Klebsiella pneumoniae*	1	5.0	1	5.0
	*Mycobacterium brisbanense*	1	5.0		
	*Mycobacterium peregrinum*			1	5.0
	*Pseudomonas stutzeri*	1	5.0		
	*Staphylococcus aureus*	3	15.0	3	15.0
	*Staphylococcus agalactiae*			1	5.0
	*Staphylococcus epidermidis*	6	30.0	8	40.0
	*Staphylococcus haemolyticus*	3	15.0	1	5.0
	*Staphylococcus hominis*	7	35.0	6	30.0
	*Staphylococcus pasteuri*			1	5.0
	*Staphylococcus saccharolyticus*	1	5.0		
	*Streptococcus agalactiae*			1	5.0
	*Streptococcus oralis*	1	5.0		
	Total species	17		9	
Control Group					
	Unidentified			2	15.4
	*Aspergillus parasiticus*	1	7.7		
	*Bacillus altitudinis*			1	7.7
	*Bacillus siamensis*			1	7.7
	*Bacillus sonorensis*	1	7.7		
	*Bacillus pumilus*			1	7.7
	*Citrobacter freundii*			1	7.7
	*Corynebacterium amycolatum*	1	7.7		
	*Enterococcus faecalis*	1	7.7		
	*Enterococcus faecium*	1	7.7		
	*Micrococcus luteus*	2	15.4		
	*Staphylococcus aureus*	1	7.7		
	*Staphylococcus epidermidis*	3	23.1	4	30.8
	*Staphylococcus haemolyticus*	2	15.4		
	*Staphylococcus hominis*	4	30.8	2	15.4
	*Staphylococcus lugdunensis*	1	7.7	2	15.4
	*Staphylococcus warneri*			1	7.7
	*Serratia marcescens*	1	7.7		
	Total species	12		8	

**Table 4:** Swab sampling from psoriatic plaques: results in relation to participant living environments.

Swab Sampling from Psoriatic Plaques		AEROBIC		ANAEROBIC	
		n	%	n	%
Urban					
	Lack of growth	1	9.1	1	9.1
	Unidentified			3	27.3
	*Acinetobacter* spp.	1	9.1		
	*Bacillus subtilis*	2	18.2		
	*Enterococcus faecium*	1	9.1		
	*Pseudomonas luteola*			1	9.1
	*Staphylococcus aureus*	1	9.1	2	18.2
	*Staphylococcus capitis*	1	9.1	1	9.1
	*Staphylococcus cohnii*	1	9.1		
	*Staphylococcus epidermidis*	3	27.3	4	36.4
	*Staphylococcus haemolyticus*			1	9.1
	*Staphylococcus hominis*	3	27.3	1	9.1
	*Staphylococcus warneri*	1	9.1		
	Total	9		6	
Rural					
	Lack of growth			1	11.1
	Unidentified	1	11.1		
	*Micrococcus luteus*	1	11.1		
	*Proteus mirabilis*	1	11.1	1	11.1
	*Staphylococcus aureus*	1	11.1	1	11.1
	*Staphylococcus capitis*	1	11.1		
	*Staphylococcus epidermidis*	3	33.3	3	33.3
	*Staphylococcus haemolyticus*			1	11.1
	*Staphylococcus hominis*	3	33.3	3	33.3
	*Staphylococcus lugdunesis*			1	11.1
	Total	6		6	

**Table 5:** Swab sampling from psoriatic plaques: differences in microbial agents between male and female participants.

Swab Sampling from Psoriatic Plaques		AEROBIC		ANAEROBIC	
		n	%	n	%
Male					
	Lack of growth	1	6.7	1	6.7
	Unidentified	1	6.7	2	13.3
	*Acinetobacter* spp.	1	6.7		
	*Bacillus subtilis*	1	6.7		
	*Micrococcus luteus*	1	6.7		
	*Proteus mirabilis*	1	6.7	1	6.7
	*Pseudomonas luteola*			1	6.7
	*Staphylococcus aureus*	1	6.7	1	6.7
	*Staphylococcus capitis*	2	13.4	1	6.7
	*Staphylococcus epidermidis*	5	33.5	5	33.5
	*Staphylococcus haemolyticus*			2	13.4
	*Staphylococcus hominis*	5	33.5	4	26.8
	*Staphylococcus lugdunesis*			1	6.7
	*Staphylococcus warneri*	1	6.7		
	Total species	9		8	
Female					
	Lack of growth			1	20.0
	Unidentified			1	20.0
	*Bacillus subtilis*	1	20.0		
	*Enterococcus faecium*	1	20.0		
	*Staphylococcus aureus*	1	20.0	2	40.0
	*Staphylococcus cohnii*	1	20.0		
	*Staphylococcus epidermidis*	1	20.0	2	40.0
	*Staphylococcus hominis*	1	20.0		
	Total species	6		2	

## Data Availability

No new data were created or analyzed in this study. Data sharing is not applicable to this article.
